# It’s Hard to Prepare for Task Novelty: Cueing the Novelty of Upcoming Tasks Does Not Facilitate Task Performance

**DOI:** 10.5334/joc.423

**Published:** 2025-01-15

**Authors:** Mengqiao Chai, Ana F. Palenciano, Ravi Mill, Michael W. Cole, Senne Braem

**Affiliations:** 1 Department of Experimental Psychology, Ghent University, Henri Dunantlaan 2, 9000 Ghent, Belgium; 2 Mind, Brain, and Behavior Research Center, University of Granada, 18011, Granada, Spain; 3 Center for Molecular and Behavioral Neuroscience, Rutgers University, Newark, NJ, 07102, USA

**Keywords:** Cognitive Control, Decision making, Executive functions, Learning

## Abstract

Rapidly learning new tasks, such as using new technology or playing a new game, is ubiquitous in our daily lives. Previous studies suggest that our brain relies on different networks for rapid task learning versus retrieving known tasks from memory, and behavioral studies have shown that novel versus practiced tasks may rely on different task configuration processes. Here, we investigated whether explicitly informing about the novelty of an incoming task would help participants prepare for different task configuration processes, such as pre-adjusting working memory gating functions. We hypothesized that if different task configuration processes can be prepared for, a pre-cue informing about the novelty of the upcoming task should lead to better task performance. Across four experiments, participants were first trained on a subset of tasks, followed by a test session in which pre-cues were provided in some blocks but not others. After comparing task performance between cued and uncued blocks, our results provided no evidence supporting the benefit of cueing for both practiced and novel tasks, suggesting that people cannot prepare for different task configuration processes in the absence of concrete task information.

## Introduction

In daily life, we often encounter novel situations that require us to use skills and knowledge previously acquired to solve new problems or tasks. For example, when going to a new laundromat to do laundry for the first time, even if we know the general procedure of doing laundry, such as putting laundry in the washing machine, using the coin slot to purchase laundry service, choosing a washing program, and adding the laundry detergent, we would probably still need some time and effort to figure out how to do each step correctly and in the right order in this particular laundromat that we never used before. We, as humans, are very good at this. Namely, we are capable of exploiting the task components that we have already learned by re-using or reorganizing them flexibly to solve new tasks that we have never encountered before. This ability has been recognized as a hallmark of human’s remarkable cognitive flexibility (Brass, Liefooghe, Braem, & De Houwer, 2017; Cole, Laurent & Stocco, 2013; Cole, Braver & Meiran, 2017).

In the cognitive control literature, it has been argued that preparing for new tasks, or less familiar tasks, typically requires a different level of cognitive control (e.g. Monsell, Yeung, & Azuma, 2000; Yeung & Monsell, 2003), and tends to benefit more from task preparation in advance (Cole et al., 2018). Novel tasks also seem to require dissociable neural dynamics to support task configuration compared to practiced tasks – where task configuration is defined as the cognitive process of forming a procedural task set in working memory to support task performance (Monsell, 2003). Previous brain imaging studies on novel task configuration observed that there were several brain regions and networks that were recruited in novel tasks, but not in practiced tasks (e.g. Ruge & Wolfensteller, 2010; Hartstra et al., 2011; Bourguignon et al., 2018; Mohr et al., 2016). Intriguingly, Cole et al. (2010) observed reversed neural dynamics within prefrontal cortex when people configure novel versus practiced tasks. A recent study (Mill & Cole, 2023) further elucidated this difference by showing that performing on novel tasks required subcortical regions to temporally bind task elements together into a conjunction, whereas implementing practiced tasks replied more on prefrontal cortex to retrieve the conjunction that was already formed in long-term memory, thus facilitating the task performance in practiced tasks. The above presented neural evidence also parallels the distinction between controlled and automatic processing proposed by Shiffrin & Schneider (1984). That is, during task configuration, practiced tasks can rely on automatic task retrieval from long term memory, whereas novel tasks have to be composed in working memory and guided by top-down control on the fly.

In the current study, we asked whether people can prepare for different task configuration processes depending on the novelty of the incoming task. Specifically, we hypothesized this can be realized by modulating an updating threshold (Dreisbach & Fröber, 2019; Goschke & Bolte, 2014). Specifically, gating control of working memory has been proposed as a key mechanism to support goal-directed decision-making by dynamically selecting the relevant information into working memory and prevent the intrusion of irrelevant information (Chatham & Badre, 2015; Kruijne, Bohte, Roelfsema & Olivers, 2021). Several influential working memory models (e.g. O’Reilly & Frank, 2006; Braver & Cohen, 2000; Botvinick, Niv & Barto, 2009) highlighted several subcortical and cortical regions that may act as gating controls to support flexible task performance. For example, both midbrain dopaminergic pathways (Braver & Cohen, 2000) and basal ganglia (O’Reilly & Frank, 2006; Hazy, Frank & O’Reilly, 2007) have been proposed to send gating signals to the prefrontal cortex for adaptive task context updating. Another model that focuses on prefrontal cortex function (Rougier et al., 2005) incorporated a gating unit within the prefrontal cortex and found that, without this gating unit, models’ performance on novel task generalization dropped significantly, highlighting the necessity of working memory gating mechanism in supporting flexible cognitive control (also see D’Ardenne et al.,2012; Badre, 2012).

With regards to task novelty, it would be adaptive to open working memory input gates to long term memory when knowing a practiced task is coming in order to facilitate subsequent task information retrieval and pattern completion, leading to faster task configuration. This hypothesis is in line with a recent working memory model (Oberauer, 2009; Oberauer et al., 2013) which claimed that increased availability of the activated representation, as is the case when working memory’s gate to long term memory is open, will render the representation in long term memory more easily being retrieved into the region of direct access, which leads to less time taken during task retrieval. At the same time, based on this framework, when configuring a task, it is also important to avoid irrelevant but active tasks in long term memory from being retrieved. In fact, this becomes critical when configuring a novel task since practiced tasks and novel tasks often have overlapping task components, which will create interference. To mitigate this potential interference, closing working memory gates to long term memory would be a better strategy when the incoming task is novel so that the task encoding would not trigger automatic retrieval of practiced task information from long term memory, thus avoiding conflicts in working memory during task configuration.

In the literature, only a handful of studies have investigated whether people can prepare for different task configuration requirements in a multi-task setting, and most of them adopted task switching paradigms. In one study, Karayanidis and colleagues (2009) devised a cued task switching paradigm with three tasks and found that, by including a cue signalling a task switch would occur without specifying which task it would switch to, participants showed enhanced accuracy compared to a condition without this cue. Therefore, it appears that participants could prepare for task reconfiguration when knowing a task switch was coming (also see Nicholson et al., 2006; Karayanidis et al., 2010; Dreisbach, Haider, and Kluwe, 2002, Aufschnaiter, Kiesel, & Thomaschke, 2021). However, like most task switching studies, only a few tasks (typically two to four) were included, and participants tended to be overly trained on all of these. Therefore, typical task switching paradigms are not suitable to investigate preparing for novel tasks. In addition, in a task switching experiment that includes only two or three tasks, it is still conceivable that participants reconfigure one or two alternative tasks specifically, even if the cue did not specify which task to switch to. Therefore, the hypothesized preparatory changes in working memory gating functions could still be confounded with specific task configuration itself.

To overcome these shortcomings, we adapted a behavioral paradigm that was typically used to study rapid task learning and implementation (e.g. Cole, Laurent & Stocco, 2013; Cole et al., 2018). In this paradigm, a relatively large amount of tasks with complex structures were created by permuting multiple task rules across three task dimensions (logic, sensory/semantic and motor). Among those tasks, only a handful of them were pretrained during a separate training session, leaving the majority of the tasks novel to participants before the test session. In this study, we modified this paradigm by including a pre-cue signalling the upcoming task novelty (novel or practiced). Since practiced tasks and novel tasks required different task configuration processes, we hypothesized that inclusion of this pre-cue can benefit participants from task preparation by pre-adjusting their working memory gating functions in accordance with the upcoming task novelty. Since this pre-cue only indicates the novelty of the incoming task without specifying the task identity, we can tease the preparation of task configuration processes apart from specific task configuration.

To test these hypotheses, we conducted a series of experiments where we provided this pre-cue, hereafter referred to as task type cue, indicating whether the incoming task will be either a practiced or a novel task, so that participants can prepare for the incoming task by pre-adjusting their working memory gating function. If this is the case, we would observe behavioral benefits, either faster RT or more accurate responses, in the blocks where this task type cue is available (i.e. the informative blocks) compared to blocks where it is not (i.e. the uninformative blocks).

As additional exploration, considering that the task type cue also suggests the need to either switch or repeat a working memory gating policy between consecutive trials (e.g., when a novel task has to be prepared after just executing either a practiced task, or a novel task), we also explored the potential benefits of task type cues in switching between practiced and novel tasks. We further predicted that novel tasks might benefit more from the task type cue than practiced tasks based on previous finding that novel tasks usually benefit more from task preparation (e,g. Cole et al., 2018).

## Experiment 1

**Figure 1:**
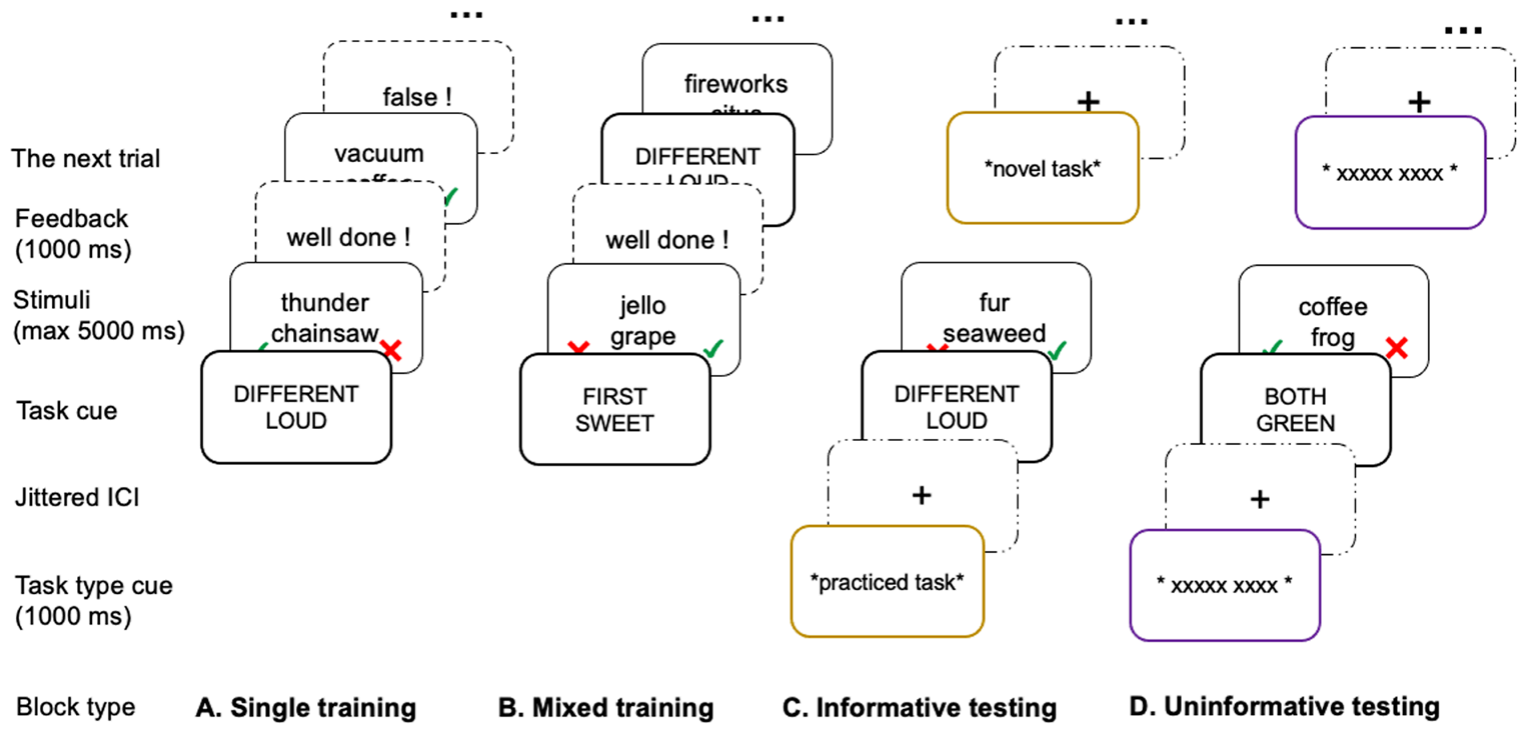
A schematic illustration of the SPRO paradigm. *Note*. The SPRO paradigm was used in both Experiment 1 and 2. Six out of thirty-six tasks were selected to be the practiced tasks that participants were trained on, during both **A**. single training and **B**. mixed training blocks. After the training session, the test session included **C**. informative blocks, in which the task type cue was provided at the beginning of each trial, specifying the incoming task being either practiced or novel, and **D**. uninformative blocks where the task type cue was replaced by an uninformative cue, which served as the control condition in order to examine the effect of task type cue. The two differences between Experiment 2 and Experiment 1 can be summarized as follows: First, the task cue was self-paced in Experiment 1, but had a fixed duration of 1000 ms in Experiment 2. Second, the response mapping of Experiment 1 varied across trials (see the red “cross” and green “tick” in the figure), but was fixed across trials for each participant in Experiment 2.

In this preregistered experiment (https://osf.io/pytzj), we designed a behavioral paradigm that we here refer to as the simplified permuted rule operations (SPRO) paradigm. In the original PRO paradigm, the experiment featured a multi-dimensional task design, which included three task dimensions, corresponding to the logical, semantic, and motor rule respectively. For each trial, the to-be-performed task was defined jointly by these three dimensions, and the subsequent two word-stimuli had to be evaluated using this multi-dimensional task rule. For example, on a given trial, the task rules could be “BOTH, SWEET, LEFT INDEX”. In this case, participants had to evaluate if the two words that followed were both sweet or not. If they were both sweet, participants had to respond with their left index finger. Otherwise, they had to respond using the other response finger of the same hand (i.e. left middle finger). By including four task rules for each task dimension, sixty-four unique tasks were created by permutating across dimensions. However, the PRO paradigm can be rather challenging for participants to perform well. In the current study, we devised a simplified version for the following reasons. First, unlike Cole et al. (2018), which mainly focused on accuracy instead of RT data, we planned to look at both RT and accuracy data. Given that a lower accuracy would decrease the power of RT analyses (i.e., because RT analyses are performed on correct trials only), we aimed for relatively high accuracy in the current study. Second, the data collection of the current study had to be performed online due to COVID restrictions. Considering that the online environment is less controlled, maximal task engagement can be less guaranteed (Clifford & Jerit, 2014), which could also result in worse performance than in-lab experiments. Taken together, we simplified the PRO paradigm by decreasing the number of task dimensions from three to two (Figure 1). Specifically, we excluded the motor dimension from the task. As an effort to still be able to create a myriad of tasks, another two task rules were added to each of the remaining task dimensions. As a result, thirty-six tasks were created by permuting these six logical and six semantic rules.

In the original PRO paradigm, four out of sixty-four tasks were selected as the practiced tasks, which participants got trained on beforehand. These four tasks also spanned all task rules across task dimensions. By doing so, each task rule would be equally trained before testing. In the current study, six tasks were selected as practiced tasks because two more task rules were added to each remaining task dimension. This way, our practiced tasks also spanned all task rules across two dimensions, leaving the remaining thirty novel tasks untrained before testing. Please note that novel tasks were novel in terms of their compositional binding structure in which participants had to combine two task dimensions in a novel way that were not practiced before. During the test session, participants were asked to perform both practiced and novel tasks in four experimental blocks. The key manipulation was that we added a task type cue at the beginning of each trial, which was either informative (specifying that the incoming to-be-performed task was either practiced or novel; see column C of Figure 1) or uninformative (a series of X’s, which equated the overall trial structure with the informative condition, but did not contain any information on the incoming task type; see column D of Figure 1). By comparing participants’ behavioral performance between informative and uninformative blocks, the utility of the task type cue (i.e. the cue informativeness) could be evaluated.

Because the benefit of task type cues could be reflected in either task preparation during task cue encoding or task performance during the decision-making stage, we made task cue encoding self-paced, meaning that participants could take as much time as they needed to prepare for a task following the task cue, and needed to press the spacebar on the keyboard to indicate that they were ready to perform the task. Therefore, there were two reaction times measured on each trial, one to encode and prepare for a task (i.e. task cue RT), and another one to make a decision and respond to the stimuli (i.e. target RT).

### Methods

#### Participants

60 participants (55 females, 4 female, 1 unreported, mean age = 19.13) were recruited, all of whom were first-year Psychology students with normal or corrected-to-normal vision who studied at Ghent University and participated in exchange for course credits. Following the preregistered data exclusion procedure described below, 51 participants remained in the data analysis (46 females, 4 males, 1 unreported, mean age = 19.12). The sample size was determined so that 3000 trials would be accumulated across participants for each data cell based on the factorial design of current study (i.e. the interaction between task novelty and cue informativeness), which was above the minimum number of observations suggested by Brysbaert & Stevens (2018). By using this design, we were able to detect a small-to-medium effect (around 0.356 in Cohen’s d) with 80% statistical power. All participants gave their informed consent before participation.

#### Tasks and Materials

There were two task dimensions with six task rules per dimension. The task rules of the logical dimension included: BOTH, AT LEAST ONE, NEITHER, FIRST, SECOND, and DIFFERENT,^1^ and the semantic rules included: GREEN, LOUD, SWEET, SOFT, SCENTED, and LIVING. The inclusion of these task rules was mainly inspired by previous studies on either rapid instructed task learning or task switching (e.g., Cole et al., 2018; Braem, 2017). For each semantic rule, there were 45 words selected in the “True” condition. For instance, the words “broccoli” and “frog” were included as stimuli in the “True” condition for the semantic rule GREEN since both objects are typically green. There were also 45 words being included in the “False” condition for each semantic rule, all of which were sampled from the words in the “True” condition of other semantic rules. For example, the words “raspberry” and “storm” were in the “False” condition of semantic rule GREEN, and these words were selected from the “True” condition of semantic rules of SWEET and LOUD, respectively. Thus, there were a total of 270 words included as stimuli in the current study. The word list was composed in part from the previous study (Cole et al, 2018), and in part by the researchers of the current study. All words were selected in English and translated into Dutch by native Dutch speakers.

#### Experimental Procedures

Participants were first asked to give informed consent and demographic information, including age, sex, and Dutch fluency. Next, they were presented with general instructions about the procedure. Following these instructions, the main experiment started with six blocks of single task training on each of the practiced tasks, with one task per block (see column A in Figure 1). As mentioned before, the six practiced tasks were selected by randomly sampling from all 36 tasks for each participant, with one constraint: that there were no overlapping rules between tasks. In each single task training block, the task cue of the practiced task was shown only once at the beginning of the block so that participants had to keep the task in mind while they progressed through the block. Following this task cue, a pair of words appeared on screen for a maximum of 5000 ms until a response was made by pressing either the left (“F”) or right key (“J”) on the keyboard, indicating either “True” or “False”. Notably, the “True” and “False” response mappings between left and right key was randomly determined across trials. That is, in one trial, the left key was pressed to indicate the “True” response. In another trial, the right key press might be needed to indicate “True” instead. On each trial, the response mapping was made clear to participants by including a red cross (as the “False” response) and a green tick (as the “True” response) on the right and left side of the lower screen (see Figure 1) together with a word pair during stimuli presentation. Performance feedback was given at the end of each trial for 1000 ms. In total, there were 60 trials per block. On each trial, the word pair was randomly sampled from the corresponding word list of the relevant semantic task rule specified by the task cue, with the constraint of having an equal amount of “True” and “False” responses within a block. Moreover, each word, once presented, was not allowed to reoccur within the next five trials to avoid potential response bias introduced by the episodic binding between certain words and responses from previous trials (Hommel, 2004). After six single task training blocks, participants needed to go through one mixed training block (see column B in Figure 1) to also practice switching between tasks. The mixed training block comprised all six practiced tasks intermixed with each other without task repetitions between consecutive trials, and each trial started with a task cue specifying the to-be-performed task. Therefore, taken together, each participant went through seven training blocks with only short breaks in between blocks. At the end of training, each participant went through 70 trials per practiced task.

The test session was conducted immediately after the training session and included four experimental blocks. Two were informative blocks (see column C of Figure 1) featuring an informative task type cue at the beginning of each trial for 1000 ms, indicating that the incoming task would be either practiced or novel. The other two blocks were uninformative blocks (see column D of Figure 1), in which the task type cue was replaced by a visually similar uninformative cue at the beginning of each trial, thus providing no information about the incoming task. As described above, the uninformative blocks were included as a control condition in comparison to the informative blocks. Across four experimental blocks, the two informative blocks were intermixed with two uninformative blocks, with the starting block type counterbalanced across participants. In each test block, the practiced tasks were intermixed with novel tasks that participants had not performed before. More specifically, half of the trials (30 trials) were practiced tasks in a balanced way (i.e. five trials per task), leaving the other 30 trials as novel tasks, with each novel task only appearing once per block, thus four times in total. The task sequence was randomly determined with no repeating semantic or logical rules between consecutive trials. There was no feedback at the end of each trial in the test session. At the end of the experiment, two additional questions were asked of participants to gauge whether they considered the practiced tasks easier than the novel tasks, as well as if they thought the task type cue was helpful or not. The inclusion of the first question was to check whether participants experienced any difficulty differences between practiced versus novel tasks. The second question was to compare participants’ answers to their actual performance to evaluate the utility of the task type cue. In total, the whole experiment took around 90 minutes. This experiment, as well as all the following experiments, were approved by the Ethical Committee of the Faculty of Psychology and Educational Sciences at Ghent University, under the title of “What makes us cognitively flexible? A new learning perspective”.

#### Data analysis

The analysis pipeline was preregistered on OSF (https://osf.io/pytzj). Raw data are also available at https://osf.io/fstn6/. All data processing and analyses were conducted using R (R Core Team, 2017). Participants whose overall accuracy was either below 70%^2^ in practice section or below 55% in the test session were removed from analyses. Next, participants whose average task cue RT, target RT, or error rate (ER) exceeded ± 2.5 standard deviations (SD) from the group mean were also discarded. Furthermore, trials following errors or with RT (on either the task cue or target) faster than 200 ms were also removed from analyses. Error trials were excluded only from RT analysis. In addition, trials with extremely long task cue RT (> 5s) were also discarded since it might indicate task disengagement. Finally, trials with RTs (either task cue or target) ±2.5 SD from the individual mean were also excluded.

Task cue RT, target RT, and target accuracy were analyzed separately using ANOVAs implemented by the “ez” R package (Lawrence, 2016). Since we are mainly interested in whether participants could benefit from the task type cue in the test session, we only included data from the test session in the ANOVAs. Specifically, in each ANOVA, the cue informativeness (informative or uninformative), as well as task novelty (practiced or novel task) were included as within-subject factors. Considering that some participants started with the informative block, while others started with an uninformative block, the starting block type was also included as a between-subject covariate (because data was collected online and participants were randomly assigned to either group, thus the counterbalancing was not fully achieved). Therefore, an ANOVA of mixed design was used to include both within-subject factors and between-subject factors. The effect size of each factor was quantified by generalized eta squared (η^2^) and reported for all statistically significant results.

In case of null result found in the main effect of task type cue or the interaction between task type cue and task novelty in the confirmatory analysis, we additionally evaluate the evidence in favor of the null hypothesis by computing the Bayes factor using the “bayesfactor” R package (Morey et al., 2015). To clarify, the Bayes Factor permits disambiguation of whether a non-significant result reflects support for the null hypothesis, or just arises from insensitivity of the statistical test (Hoijtink, Mulder, van Lissa, & Gu, 2019). Thus, we additionally computed Bayes factor for the non-significant results from the ANOVA analysis as an effort to quantify the evidence supporting the null hypothesis, as reflected by BF_01._ The interpretation follows what Jeffreys (1998) proposed, as follows: BF = 1 - No evidence; 1 < BF < = 3 – Anecdotal; 3 < BF < = 10 – Moderate; 10 < BF < = 30 – Strong; 30 < BF < = 100 - Very strong; and BF > 100 - Extreme. Please note that these additional analyses on the Bayes factor are reported as supplementary results that we do not draw conclusion on to keep our inference system consistent, as suggested by Dienes (2024). Thus, please interpret the results of Bayes factors with caution. The frequentist statistics, as implemented by ANOVAs, are the inference system that we used to interpret the data and draw conclusion on, as pre-registered.

In addition to testing the benefit of task type cue, we also conducted exploratory analysis regarding the potential benefit of task type cues in switching between practiced and novel tasks. To this end, we conducted another ANOVA with an additional within-subject factor of the trial type sequence (repeat or switch). Any significant interaction including trial type sequence and cue informativeness was followed by post-hoc comparisons using the Bonferroni–Holm method to adjust for multiple comparisons.

### Results

**Figure 2:**
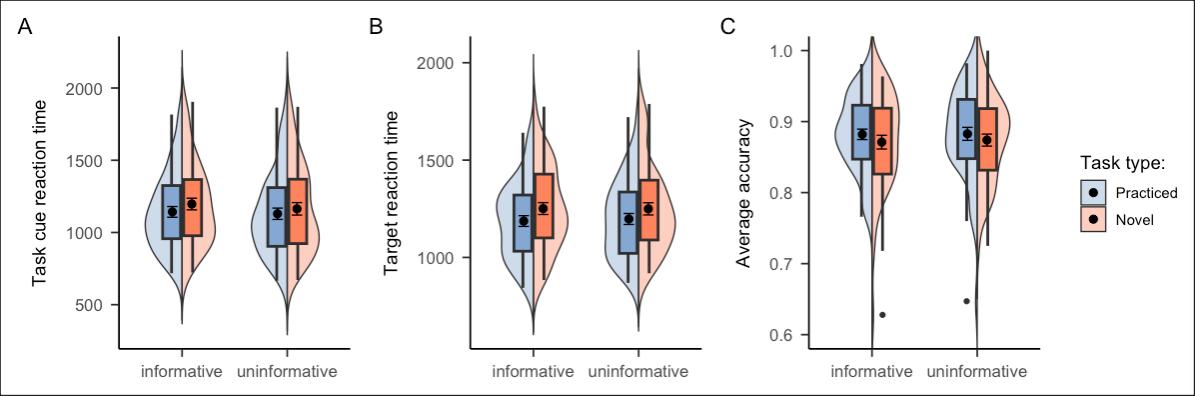
Mean RTs and accuracy of Experiment 1 as a function of cue informativeness and task novelty. *Note*. The results of Experiment 1 for **A**. task cue RT, **B**. target RT, and **C**. target accuracy, respectively. The black points within the box plots denote the mean, and the error bar denote standard errors.

We first examined the task cue RT. A significant main effect of task novelty was observed (see Panel A of Figure 2), with practiced tasks (*M* = 1136.89, *SE* = 37.35) being encoded and prepared significantly faster than the novel tasks (*M* = 1179.60, *SE* = 40.93), *F*(1, 49) = 20.25, *p* < .001, *η^2^* = .006. However, task encoding and preparation in the informative blocks (*M* = 1169.91, *SE* = 38.35) did not benefit from the task type cue compared to uninformative blocks (*M* = 1146.55, *SE* = 40.54), *F*(1, 49) = 3.44, *p* = .069, *BF_01_* = 16.21, challenging the utility of the task type cue in task preparation. In fact, participants’ reaction times were numerically higher in informative compared to uninformative blocks. The interaction between task type cue and task novelty also did not reach statistical significance, *F*(1, 49) = 1.65, *p* = .205, *BF_01_* = 13.22. The corresponding target RT analysis showed a similar pattern. A significant main effect of task novelty was observed (see Panel B of Figure 2), with practiced tasks (*M* = 1191.96, *SE* = 26.91) being responded significantly faster than the novel tasks (*M* = 1250.02, *SE* = 29.91), *F*(1, 49) = 44.18, *p* < .001, *η^2^* = .02. Again, no benefit of task type cue was observed when comparing RT between informative and uninformative blocks, *F* < 1, *BF_01_* = 4.51. The interaction between task type cue and task novelty was also non-significant, *F* < 1, *BF_01_* = 10.25. In terms of accuracy (see Panel C of Figure 2), neither task novelty, *F*(1, 49) = 1.89, *p* = .175, nor task type cue, *F* < 1, *BF_01_* = 8.23, yielded significant results, although the accuracy of practiced tasks (*M* = 0.88, *SE* = 0.01) was indeed numerically higher than novel tasks (*M* = 0.87, *SE* = 0.01). The interaction between task type cue and task novelty was again non-significant, *F* < 1, *BF_01_* = 7.54. In addition, we explored whether the task type cue would help participants switch between the different task types (i.e., practiced and novel tasks). However, we did not find any significant results of trial type sequence (either repeat or switch). Therefore, taken together, there was no evidence supporting a potential benefit of task type cue in preparing or performing the incoming task, or switching between trial types. The cell means and corresponding standard deviations of each experimental condition are presented in Table 1.

**Table 1:** The cell means and standard errors of RT and accuracy data in Experiment 1 as a function of cue informativeness and task novelty.


MEASURES	*INFORMATIVE*				*UNINFORMATIVE*			
	
	*PRACTICED*		*NOVEL*		*PRACTICED*		*NOVEL*	
			
	*M*	*SD*	*M*	*SD*	*M*	*SD*	*M*	*SD*

Task cue RT	1,142.61	263.86	1,196.95	288.46	1,129.62	280.08	1,163.62	308.05

Target RT	1,186.86	195.68	1,250.99	217.78	1,197.55	201.00	1,249.48	223.00

Accuracy	0.88	0.05	0.87	0.07	0.88	0.07	0.87	0.06


*Note*. RT = reaction time; M = mean; SD = standard deviation.

The questionnaire collected at the end of experiment revealed that, out of fifty-one participants, only four people reported the task type cue as “mildly helpful”. In fact, three of these four participants started with uninformative blocks, which could explain why the task type cue appeared helpful because the informative blocks could be experienced as easier than uninformative blocks due to a practice effect.

### Discussion

The result of Experiment 1 replicated the novelty cost demonstrated by previous studies (e.g. Cole et al., 2018) that novel tasks took longer to prepare and respond to than practiced tasks. Critically, we extended this novelty cost to both task preparation stage and task implementation stage, evidenced by significant slowing-down in both task cue RT and target RT for novel tasks. Previous studies typically used the target RT as the indicator of time needed to both prepare and implement the task, thus any behavioral effect could be attributed to either or both stages. Here, by making the task cue self-paced, we clearly showed that task preparation, without the possible confound of task implementation, tended to be more demanding if the task was a novel one rather than a practiced one.

However, we found no evidence supporting our main hypothesis regarding the behavioral benefit of task type cue, evidenced by the null results of cue informativeness in all analysis presented above. Therefore, our findings cast doubt on whether participants were able to adjust their working memory gating function in advance when facing tasks that required different task configuration processes.

However, there were some caveats in the experimental design that could contribute to our null findings. First, as described above, the response mapping, which determined which key on the keyboard participants should use for the “True” and “False” responses was determined randomly across trials. In addition, the response mapping was shown with the word-stimuli, which means that participants did not know whether they should press either the left or right key to indicate the “True” response until the stimuli appeared on the screen. By doing so, it might have discouraged participants from preparing for the task in advance since the response mapping was uncertain until stimuli onset. If this were the case, then it is conceivable that participants would also not make use of the task type cue to prepare in advance since advanced task preparation was, in general, discouraged. Moreover, in Experiment 1, participants had to respond to both task cue and stimuli, thus generating two responses per trial. The reason for this design choice was to be able to ascribe the potential benefit of task type cue to either (or both) the task encoding or (or and) task implementation stage. However, the downside of this design can be that, by responding to both task cue and stimulus, the potential effect of task type cue spread out across these two task stages, thereby decreasing the power of detecting the effect of task type cue. Therefore, in our next experiment, we modified the design to address these two concerns.

## Experiment 2

In Experiment 2, we aimed to address the potential caveats of Experiment 1 by introducing two modifications. First, because introducing variation in response mapping across trials could risk discouraging participants to prepare for the incoming task, we fixed the response mapping throughout the experiment for each participant in Experiment 2. By doing so, participants were encouraged more to prepare for the task in advance. Second, instead of being self-paced in Experiment 1, we introduced a limited duration of task cue as an effort to push any potential benefit of the task type cue into the task implementation stage and avoid the possibility of diluting the effect across multiple task stages.

### Methods

#### Participants

52 first-year Psychology students (40 females, 12 males, mean age = 18.92) who studied at Ghent University were recruited in exchange for course credits. Following the same data exclusion procedure described above, 47 participants remained in the data analysis (35 females, 12 males, mean age = 18.87). This sample size was comparable to Experiment 1. All participants gave their informed consent prior to their participation.

#### Experimental procedures

The experimental structure, procedure, as well as materials were exactly the same as Experiment 1, except for two modifications. First, the duration of task cues was fixed to 1000 ms (as in Cole et al., 2018) instead of being self-paced. Second, the response mapping of “True” and “False” responses was fixed for each participant, yet counterbalanced across participants. Similar to Experiment 1, participants started with six single training blocks and one mixed training block on six practiced tasks before moving on to the test session, which included two informative blocks with task type cues, as well as two un-informative blocks with uninformative task type cues. Our main interest was still to examine if participants were able to use the task type cues to help them prepare for the incoming task.

### Results

**Figure 3:**
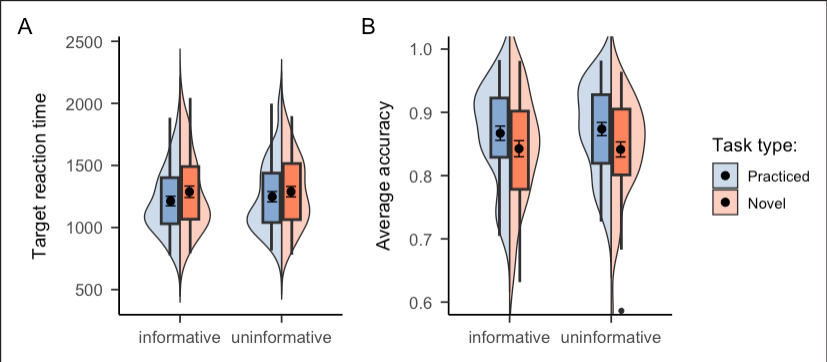
Mean RTs and accuracy of Experiment 2 as a function of cue informativeness and task novelty. *Note*. The black points within the box plots denote the mean, and the error bar denote standard errors.

Our analysis again focused on the test session. A significant main effect of task novelty was observed in target RT, *F*(1, 44) = 25.41, *p* < .001, *η^2^* = .01, showing that participants responded significantly faster on practiced tasks (*M* = 1230.51, *SE* = 38.67) compared to novel tasks (*M* = 1287.13, *SE* = 42.49), as shown in Panel A of Figure 3. However, no significant benefit of task type cue was observed, *F*(1, 44) = 1.72, *p* = .196, *BF_01_* = 1.96, although target RT of informative blocks (*M* = 1250.10, *SE* = 41.28) was numerically lower than uninformative blocks (*M* = 1267.11, *SE* = 40.41). The interaction between task type cue and task novelty also did not reach statistical significance, *F*(1, 44) = 2.73, *p* = .106, *BF_01_* = 15.54. In terms of accuracy, unlike Experiment 1, this time we observed a significant main effect of task novelty in accuracy (see Panel B of Figure 3), *F*(1, 44) = 10.87, *p* = .002, *η^2^* = .03, with higher accuracy on practiced tasks (*M* = 0.87, *SE* = 0.01) compared to novel tasks (*M* = 0.84, *SE* = 0.01). Nonetheless, no benefit of task type cue was observed, *F* < 1, *BF_01_* = 8.07. The interaction between task type cue and task novelty was also non-significant, *F* < 1, *BF_01_* = 3.61. Cell means and corresponding standard errors of each experimental condition are presented in Table 2.

**Table 2:** The cell means and standard errors of RT and accuracy data in Experiment 2 as a function of cue informativeness and task novelty.


MEASURES	*INFORMATIVE*				*UNINFORMATIVE*			
	
	*PRACTICED*		*NOVEL*		*PRACTICED*		*NOVEL*	
			
	*M*	*SD*	*M*	*SD*	*M*	*SD*	*M*	*SD*

Target RT	1,213.16	260.66	1,287.68	313.04	1,248.13	282.01	1,288.69	284.01

Accuracy	0.87	0.08	0.84	0.09	0.87	0.07	0.84	0.08


*Note*. RT = reaction time; M = mean; SD = standard deviation.

Similar to Experiment 1, the trial type sequence (switch or repeat) was included as an additional within-subject factor in the ANOVAs in order to explore the potential benefit of task type cue in switching between practiced and novel tasks. However, we again did not find any significant effect of trial type sequence.

The questionnaire revealed that there were only four participants who reported that task type cue was “mildly helpful”, all of whom started the test session with an uninformative block. Thus, similar to Experiment 1, participants’ impression of task type cue being helpful might be due to the fact that they started with the uninformative block, thus the informative blocks tended to come across as easier due to practice effect.

### Discussion

Our motivation for conducting Experiment 2 was to re-examine the potential benefits of task type cue by using a modified experimental design. However, we still did not find evidence supporting our hypothesis of a beneficial effect of task type cue on behavior. On the other hand, we again found a robust novelty cost. In Experiment 2, when participants only had 1000 ms to encode and prepare for the task cue, we observed a novelty cost in both target RT and accuracy. In comparison, in Experiment 1, where participants could take as much time as they wanted to prepare for the task cue, the novelty cost was observed only in target RT, but not accuracy. This suggests that when participants could spend more time encoding and preparing for a novel task, they were able to perform as accurately as they do on the practiced task. It also indicates that novel tasks did need more time to prepare, as Cole et al. (2018) demonstrated. Nonetheless, the task type cue again turned out to be unhelpful.

In our next experiment (Experiment 3), we aimed to make a final attempt to make the task type cues more useful.

## Experiment 3

**Figure 4:**
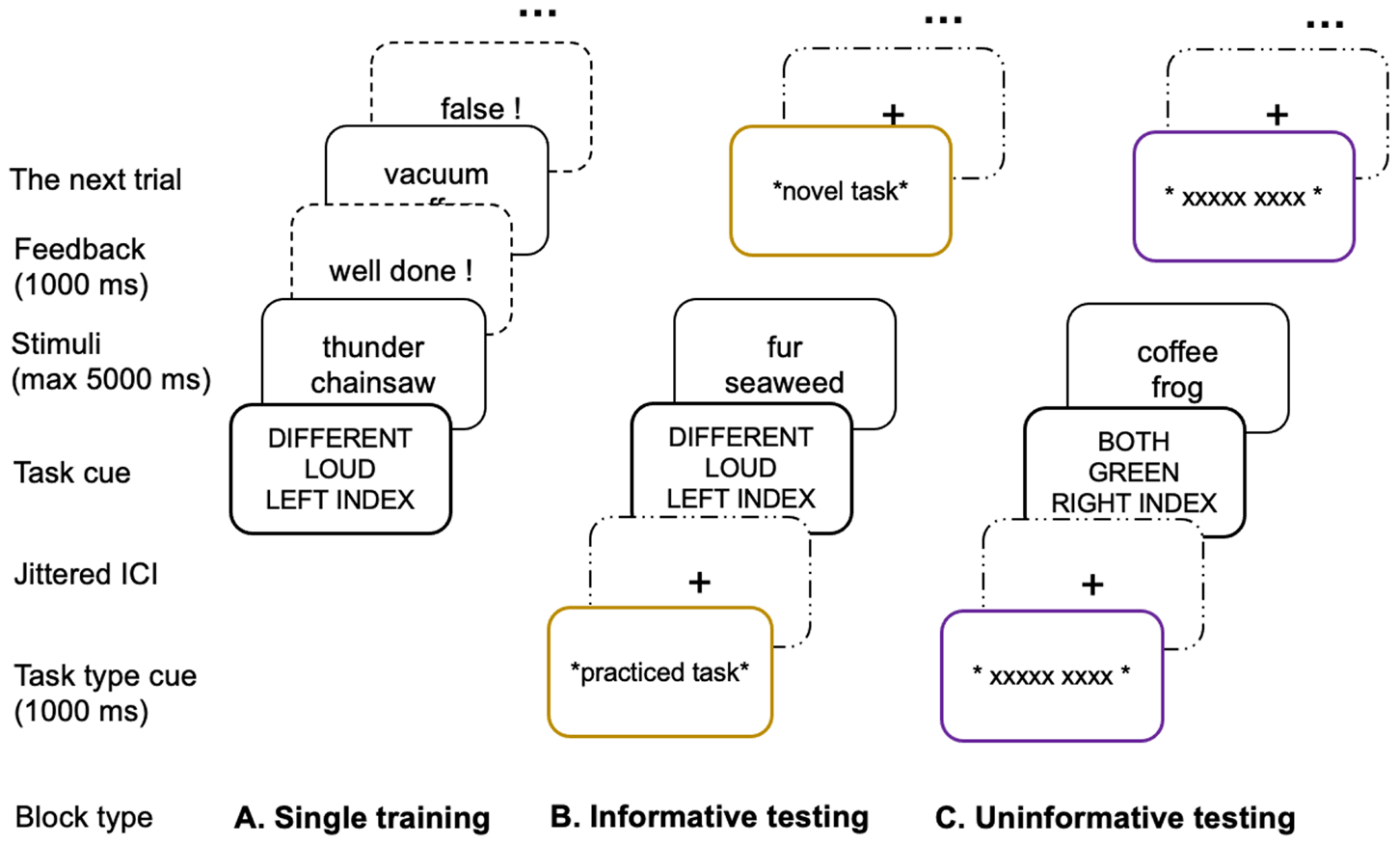
A schematic illustration of the PRO paradigm. *Note*. The PRO paradigm was used in both Experiment 3 and 4. The PRO paradigm included 64 tasks in total, 4 of which were selected to be the practiced tasks that participants went through training in the **A**. single training session. Following the training session, the test session consisted of two types of blocks: **B**. informative blocks, in which a task type cue was presented at the beginning of each trial, indicating whether the task was one of the practiced tasks or a novel task, and **C**. uninformative blocks, where the task type cue was replaced by an uninformative cue. This uninformative cue served as a control condition to investigate the impact of the task type cue. Experiment 4 had the exact same design as Experiment 3.

In the preregistered Experiment 3(https://osf.io/8z53n), we set out to further encourage people to differentially prepare for novel versus practiced tasks so that the task type cue would more likely be helpful. To this end, two new modifications were introduced. First, we aimed to further distinguish practiced tasks from novel tasks in terms of task space (Kleinsorge & Heuer, 1999). Here the task space refers to the amount of tasks from which the task of a certain trial can be selected. Since the task of each trial is randomly chosen with some additional constraints (see experimental procedures of the method section of Experiment 1), the task space was also informative in terms of uncertainty level. From the perspective of probability theory, a larger task space corresponds to more uncertain environment with less predictability in terms of which task will be selected. In the original PRO paradigm, there were four practiced tasks and sixty novel tasks. In comparison, in our previous two experiments, we had six practiced tasks and thirty novel tasks. Therefore, in terms of task space, there was a larger difference between practiced and novel tasks in the original paradigm (4/64 tasks were practiced) compared to ours (6/36 tasks were practiced), which also means that there is a larger difference in uncertainty level between practiced tasks and novel tasks in terms of task identity in the original design. This difference in task uncertainty could impact participants’ preparatory strategy since uncertainty level is indicative of the need for flexibility, which is a key factor in modulating updating thresholds (Dreisbach & Fröber, 2019). Thus, increasing the difference in task space between practiced and novel tasks could be one solution that might encourage participants to make better use of the task type cue. Therefore, in Experiment 3, we adopted the original design of the PRO paradigm (e.g. Cole et al., 2018) by adding the motor task rule back to the experiment (see Figure 4). By doing so, each task included three task dimensions with four task rules per dimension, thus creating 64 different tasks.

In addition to increasing the difference in task space between practiced and novel tasks, we strived to further differentiate the task configuration processes between practiced and novel tasks. Making the practiced tasks more familiar and the novel tasks more unfamiliar to participants could be another approach to make the task type cue more useful since it would make the task configuration process of practiced tasks rely more on long term memory retrieval and novel tasks on working memory updating. To this end, we increased the amount of training for practiced tasks in the training session, while decreasing the amount of exposure to novel tasks in the test session. By doing so, we further encouraged participants to adopt diverging task configuration processes between these two task types, thereby making the task type cue more useful for them to adjust their working memory gating function in advance.

### Methods

#### Participants

66 participants (29 females, 37 males, mean age = 28.73) were recruited on Prolific (https://www.prolific.co/), all of whom were between 19–36 years old and spoke English as their first language. The participation was compensated for £ 9.34. After the data exclusion procedure specified above, data from 56 participants remained (24 females, 32 males, mean age = 28.79) for analysis. All participants gave their informed consent prior to their participation.

#### Tasks and materials

The first major modification on the task was to include the motor rules. By doing so, each task was comprised of three task dimensions (i.e. logical, semantic, and motor rules), with four possible task rules per dimension. The motor rule could be one of the following: LEFT MIDDLE, LEFT INDEX, RIGHT MIDDLE, RIGHT INDEX. These four motor rules correspond to key pressing of “D”, “F”, “J”, and “K” on the keyboard respectively. For each trial, participants had to categorize the stimuli based on the logical and semantic rules, as in Experiment 1 and 2. However, instead of always pressing one of the two keys, as in Experiments 1 and 2, to indicate the correct response, now it could be one of four keys, which was specified by the motor rule, and spacebar needed to be pressed to indicate the “incorrect” response. The logical rules included BOTH, AT LEAST ONE, FIRST, AND SECOND, and the semantic rules included GREEN, LOUD, SWEET, AND SOFT. Since the experiment was conducted on Prolific, all task rules and stimuli words were presented in English, instead of Dutch.

#### Experimental procedures

The experiment started with a training session. As stated above, we extended the training session for each of the four practiced tasks. In the previous two experiments, the training session included seventy trials for each practiced task. In Experiment 3, participants would, instead, go through two training blocks with 60 trials per block, for each practiced task, thus almost doubling the amount of training per practiced task. Given that there were four practiced tasks, a total of eight training blocks was included in the training session. The test session included four blocks, with each block containing 30 trials of novel tasks and 30 trials of practiced tasks. Considering the fact that there were 60 novel tasks in Experiment 3, each novel task would appear only twice throughout the whole test session, in comparison to the previous two experiments, where participants encountered each novel task four times. Apart from the above-mentioned differences, the rest of the procedures were exactly the same as Experiment 2.

### Results

**Figure 5:**
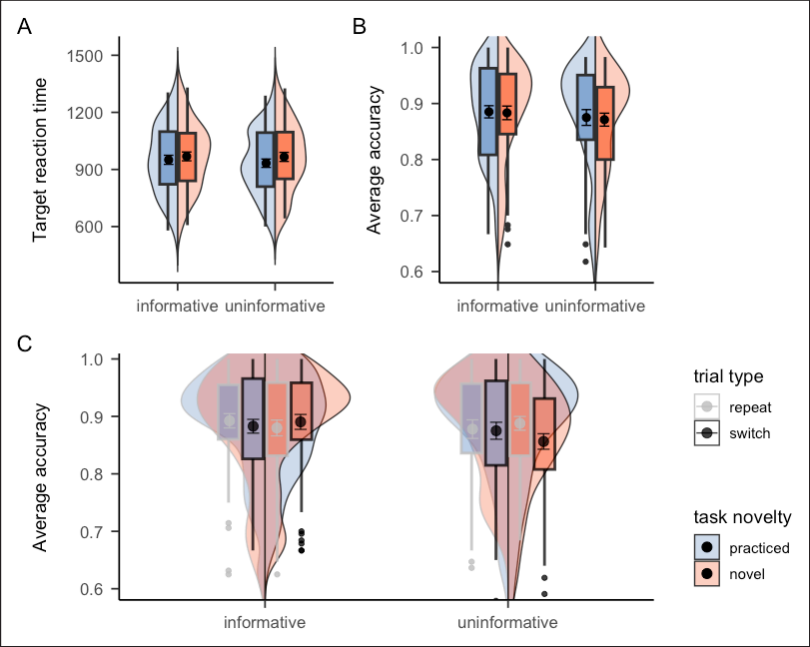
Mean RTs and accuracy of Experiment 3 as a function of cue informativeness, task novelty, and trial type. *Note*. The results for Experiment 3 in **A**. target reaction time and **B**. accuracy without considering the effect of trial type sequence. In **C**. accuracy is presented separately depending on the trial type sequence being either a repeat or switch trial in terms of task novelty. The black points within the box plots denote the mean, and the error bars denote standard errors.

A significant main effect of task novelty was observed in target RT, *F*(1, 54) = 24.37, *p* < .001, *η^2^* = .01, reflected in faster reaction for practiced tasks (*M* = 942.88, *SE* = 22.45) compared to novel tasks (*M* = 966.51, *SE* = 22.89), as shown in Panel A of Figure 5. However, there was no evidence showing a benefit of task type cue, *F*(1, 54) = 1.72, *p* = .196, *BF_01_* = 13.84, with RT of informative blocks (*M* = 959.82, *SE* = 23.13) being numerically larger than uninformative blocks (*M* = 950.48, *SE* = 22.62). The interaction between task type cue and task novelty was also non-significant, *F*(1, 54) = 1.29, *p* = .261, *BF_01_* = 2.18. Meanwhile, neither task novelty, *F* < 1, nor cue informativeness, *F*(1, 54) = 2.43, *p* = .125, *BF_01_* = 1.45, reached significance in the accuracy data (see Panel B of Figure 5). The interaction between task type cue and task novelty was again non-significant, *F* < 1, *BF_01_* = 6.12. Cell means and corresponding standard errors of each experimental condition are presented in Table 3.

**Table 3:** The cell means and standard errors of RT and accuracy data in Experiment 3 as a function of cue informativeness and task novelty.


MEASURES	*INFORMATIVE*				*UNINFORMATIVE*			
	
	*PRACTICED*		*NOVEL*		*PRACTICED*		*NOVEL*	
			
	*M*	*SD*	*M*	*SD*	*M*	*SD*	*M*	*SD*

Target RT	950.74	178.73	968.71	173.47	932.97	165.07	965.99	177.83

Accuracy	0.88	0.08	0.88	0.09	0.88	0.11	0.87	0.09


*Note*. RT = reaction time; M = mean; SD = standard error.

However, additional exploratory analyses including trial type sequence in the ANOVA revealed a potential benefit of task type cue in switching between practiced and novel tasks. Specifically, there was a significant three-way interaction between task novelty, cue informativeness, and trial type sequence, *F*(1, 54) = 6.09, *p* = .017, *η^2^* = .004 in the accuracy data (see Panel C of Figure 5). Since we hypothesized that novel tasks might benefit more from task type cue than practiced tasks, we further conducted post-hoc analysis which specifically examined the interaction between cue informativeness and trial type sequence separately for practiced and novel tasks. The result revealed a significant interaction between cue informativeness and trial type sequence for novel tasks, *F*(1, 54) = 9.12, *p* = .004, *η^2^* = 0.012. This significant interaction demonstrated that, in uninformative blocks, repeating novel trials (*M* = 0.89, *SE* = 0.01) tended to be more accurate than switching from a practiced task to a novel task (*M* = 0.86, *SE* = 0.01), *p* = .045. However, this difference was not significant, *p* = 1, when the task type cue was provided, suggesting that participants could make use of the task type cue to help them switch from practiced tasks to novel tasks. However, the interaction between cue informativeness and trial type sequence was not significant for practiced tasks, *F* < 1. This insignificant interaction suggested that facilitating effect of task type cue was not evident when switching from novel tasks to practiced tasks, suggesting that task type cue was particularly helpful when participants switched from practiced to novel tasks, as reflected in accuracy for practiced-novel tasks being less distinguishable from novel-novel tasks in the informative compared to uninformative blocks. None of the other trial type sequence related interactions, including the interaction with cue informativeness, *F*(1, 54) = 2.16, *p* = .147, *η^2^* = .002, as well as the interaction with task novelty, *F* < 1, are significant. In the meantime, there was no significant effect regarding the trial type sequence in the RT result.

In the questionnaire at the end of the experiment, four participants reported the task type cue as either “mildly helpful” or “very helpful”, all of whom started the test session with uninformative blocks, which again likely reflected a practice effect.

Taken together, the behavioral data from Experiment 3 indicated that the inclusion of task type cue did not help people perform better in either practiced or novel tasks. However, when switching from practiced to novel tasks, information regarding the type of incoming task (novel tasks in this case) seemed to help participants perform more accurately.

### Discussion

In Experiment 3, we further modified the experimental design as an effort to distinguish the task configuration processes between practiced and novel tasks. However, there was again no clear evidence supporting the benefit of task type cues on performing either practiced or novel tasks. However, the results suggested that task type cue might help participants when switching from practiced to novel tasks, suggesting that working memory gating functions could be adjusted in advance when switching from practiced to novel tasks.

Importantly, the switch-specific effect of task type cues was observed in an exploratory analysis that was not preregistered. Therefore, we wanted to see further whether this result could be replicated in an independent dataset. Therefore, for our next pre-registered experiment, we collected another group of participants using the same experimental design with the purpose of confirming whether the facilitating effect of task type cue on switching to novel tasks was reliable or not.

## Experiment 4

The aim of Experiment 4 was simply to replicate the behavioral result of Experiment 3 to make sure these findings were reliable. Therefore, the experimental design of Experiment 4 was the same as Experiment 3, as preregistered in OSF (https://osf.io/a3gqc). Specifically, the effect of interest in this replication experiment was the 3-way interaction effect between task novelty, cue informativeness, and trial type sequence found in the exploratory analysis of Experiment 3. This significant result suggests that participants could make use of task type cue to switch from practiced tasks to novel tasks, but not vice versa. Given the exploratory nature of this analysis, we aimed to try replicating this result in a separate data collection first before drawing any conclusion on it.

### Methods

#### Participants

A total of 80 participants (37 females, 40 males, 1 other, 2 unreported, mean age = 28.99) were recruited from the Prolific platform (https://www.prolific.co/). All of these participants fell within the age range of 19 to 36 years and had English as their primary language. They received £9.34 as compensation for their participation. Following the aforementioned data exclusion process, 68 participants were retained (31 females, 34 males, 1 other, 2 unreported, mean age = 28.94) for subsequent data analysis. All participants willingly provided informed consent before taking part in the study.

### Results

**Figure 6:**
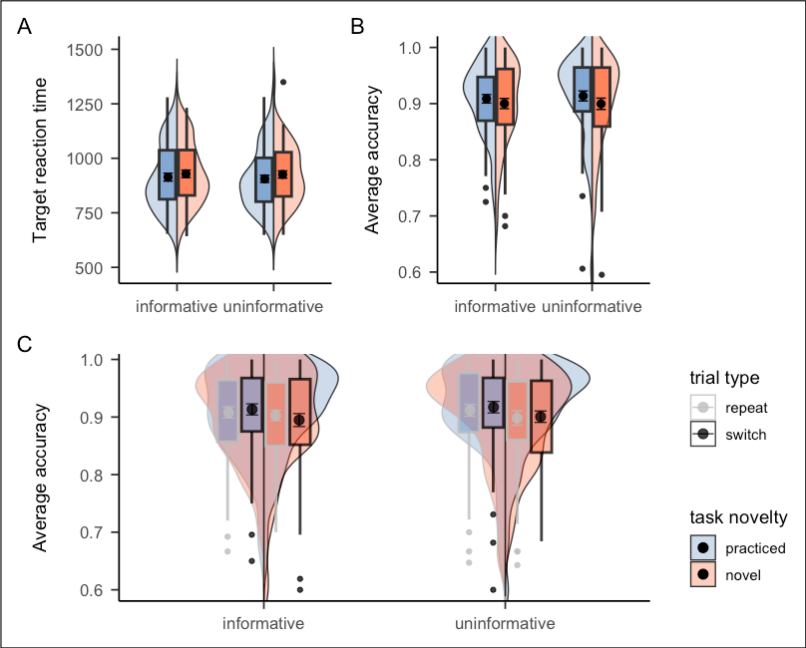
Mean RTs and accuracy of Experiment 4 as a function of cue informativeness, task novelty, and trial type. *Note*. The results of **A**. target reaction time and **B**. accuracy rate do not distinguish the trial type sequence, whereas the result of **C**. accuracy rate is presented separately depending on the trial type sequence being either a repeat or switch trial. The black points within the box plots denote the mean, and the error bar denote standard errors.

The RT results revealed a significant main effect of task novelty, *F*(1, 66) = 11.66, *p* = .001, *η^2^* = .003, showing that participants responded significantly faster in practiced tasks (*M* = 909.32, *SE* = 17.17) compared to novel tasks (*M* = 927.26, *SE* = 16.48), as shown in Panel A of Figure 6. However, no significant effect of task type cue was observed, *F*(1, 66) = 2.14, *p* = .149, *BF_01_* = 13.55, with RT of informative blocks (*M* = 921.01, *SE* = 17.43) being numerically higher than uninformative blocks (*M* = 915.73, *SE* = 16.36). The interaction between task type cue and block type was non-significant as well, *F* < 1, *BF_01_* = 4.54. In terms of accuracy, there was a significant main effect of task novelty (see Panel B of Figure 6), *F*(1, 66) = 4.78, *p* = .032, *η^2^* = .006, with higher accuracy in practiced tasks (*M* = 0.91, *SE* = 0.01) compared to novel tasks (*M* = 0.90, *SE* = 0.01). However, the accuracy results did not show any benefit of task type cue, *F*(1, 66) = 1.09, *p* = .301, *BF_01_* = 9.82. The interaction between task type cue and block type was also non-significant, *F* < 1, *BF_01_* = 4.61. Cell means and corresponding standard errors of each experimental condition are presented in Table 4.

**Table 4:** The cell means and standard errors of RT and accuracy data in Experiment 4 as a function of cue informativeness and task novelty.


MEASURES	*INFORMATIVE*				*UNINFORMATIVE*			
	
	*PRACTICED*		*NOVEL*		*PRACTICED*		*NOVEL*	
			
	*M*	*SD*	*M*	*SD*	*M*	*SD*	*M*	*SD*

Target RT	913.46	149.97	928.73	141.74	905.86	137.83	925.75	136.69

Accuracy	0.91	0.06	0.90	0.07	0.91	0.07	0.90	0.08


*Note*. RT = reaction time; M = mean; SD = standard deviation.

As the main purpose of Experiment 4, we again included trial type sequence as another within-subject factor in the ANOVA to see if the benefit of task type cue when switching to novel tasks, as observed in Experiment 3, can be replicated in Experiment 4 or not. For the target RT, there was no significant results regarding trial type sequence. In terms of accuracy, unlike what we observed in Experiment 3, we did not find any significant effect regarding trial type sequence. The critical three-way interaction between task novelty, cue informativeness, and trial type sequence, which was significant in Experiment 3, was not significant in Experiment 4 (see Panel C of Figure 6), *F* < 1. Combining data from both Experiment 3 and 4 also did not yield a significant interaction, *F*(1, 122) = 1.42, *p* = .236. Thus, although we used the exact the same design as Experiment 3, we did not replicate the significant interaction effect involving task type cue in Experiment 4.

There were seven participants who reported the task type cue being “mildly helpful” or “very helpful”, six of whom started the test session with the uninformative blocks, which again mostly likely reflected a practice effect.

### Discussion

Our aim of Experiment 4 was to replicate the finding of Experiment 3 regarding the effect of task type cue on switching to novel tasks. However, we did not replicate this result, suggesting that the effect we observed in Experiment 3 was not reliable.

## Meta-analysis across experiments

**Figure 7:**
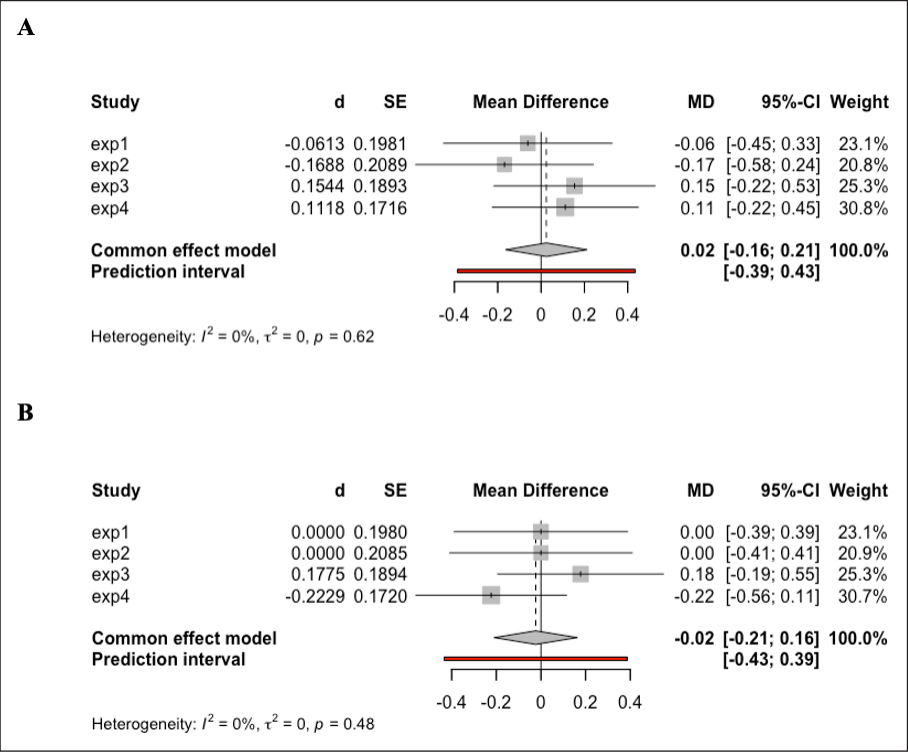
The results of meta-analysis regarding the main effect of task type cue in RT and accuracy across all experiments. *Note*. **A**. Results for RT. Negative mean difference indicates faster RT in informative compared to non-informative blocks. **B**. Results for accuracy. Positive mean difference indicates higher accuracy in informative compared to non-informative blocks.

**Figure 8:**
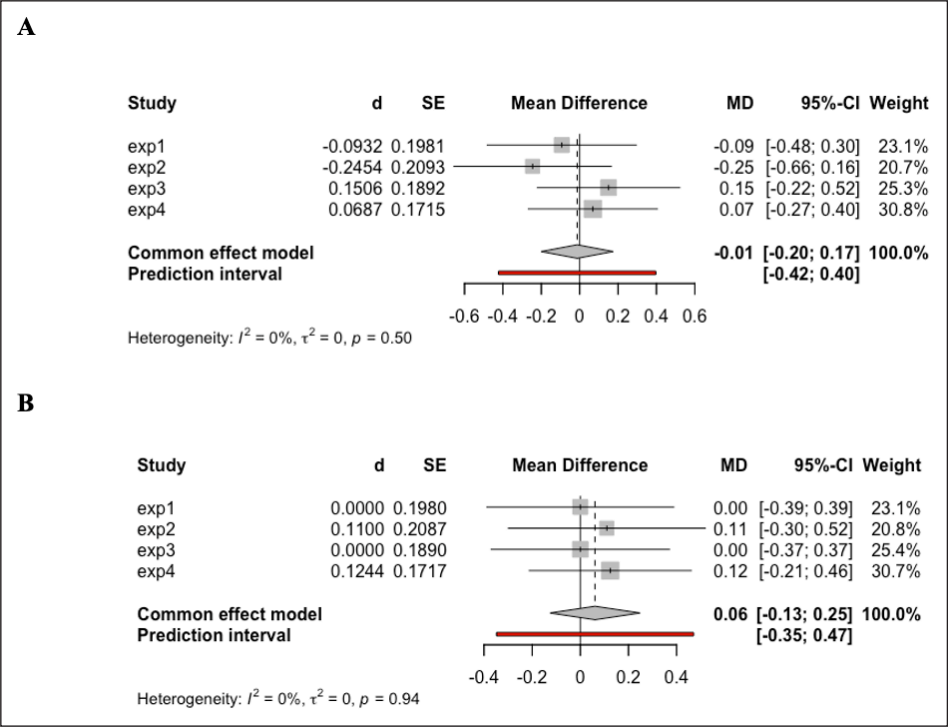
The results of meta-analysis regarding the interaction between cue informativeness and task novelty in RT and accuracy across all experiments. *Note*. **A**. Results for RT. Positive mean difference indicates greater difference in RT between informative and non-informative blocks for novel tasks compared to practiced tasks. **B**. Results for accuracy. Positive mean difference indicates greater difference in accuracy between informative and non-informative blocks for novel tasks compared to practiced tasks.

In addition to the separate analyses for each experiment, we also conducted additional meta-analyses regarding the main effect of cue informativeness and the interaction between cue informativeness and task novelty across the four experiments presented above as an effort to increase our statistical power (Cohn & Becker, 2003). We used the fixed effect model from the “meta” R package (Schwarzer, Carpenter, & Rücker, 2015) to pool effect sizes, which is quantified by Cohen’s d (*d*), across all four experiments. As shown in Panel A of Figure 7, the meta-analysis of RT results revealed no significant effect of task type cue, *d* = 0.02, 95% CI [–0.16, 0.21], z = 0.25, *p* = .799. Likewise, the result of accuracy (see Panel B of Figure 7) also demonstrated a null effect of task type cue, *d* = –0.02, 95% CI [–0.21, 0.16], z = –0.25, *p* = .806. Similarly, the interaction between cue informativeness and task novelty demonstrated null effect in both RT (see Panel A of Figure 8), *d* = –0.01, 95% CI [–0.20, 0.17], z = –0.14, *p* = .891, and accuracy (see Panel B of Figure 8), *d* = 0.06, 95% CI [–0.13, 0.25], z = 0.64, *p* = .521. The consistent null results from the meta-analysis further confirmed the lack of evidence supporting the behavioral benefit afforded by the task type cue.

## General discussion

The main objective of the current study was to investigate the extent to which people can engage in anticipatory task preparation for an incoming task based on cued information regarding task novelty without specifying task identity. To this end, we adopted a paradigm that enables us to create a myriad of tasks that differed on the level of proficiency (practiced versus novel tasks). By including a pre-cue (i.e. task type cue) indicating that the incoming task would be either a practiced or novel task, we hypothesized that participants would use this pre-cue to adjust their working memory gating functions in advance to prepare for the incoming task configuration, which would be reflected in a cueing benefit. However, across four experiments, we did not observe any benefit of this task type cue, therefore providing no evidence for the idea that participants can prepare for different types of task configuration processes.

As discussed above, some pre-cue benefits have been shown in previous studies using more limited task switching paradigms (e.g. Nicholson et al., 2006; Karayanidis et al., 2009). However, in these task switching studies, it was difficult to attribute the cueing effect solely to pre-adjusting switch readiness because task-specific strategies remained available. For example, in Karayanidis et al. (2009), the cueing benefit was found when providing a pre-cue indicating an incoming task switch without specifying which task to switch to. However, this cueing benefit most likely reflected the inhibition of the previous task instead of upregulating switch readiness in a non-specific way, evidenced by the early cue-locked positivity in the ERP data. In other words, participants likely chose to inhibit the irrelevant task instead of preparing in a task-agnostic manner for task switching. The same argument applies to other task switching studies (e.g. Nicholson et al., 2006; Liu & Yeung, 2020). In comparison, in the current study, two groups of tasks were created, namely practiced and novel tasks. Importantly, both task groups involved multiple tasks, and there was no instance of task repetitions, which means inhibiting the previous task was always beneficial regardless of task type cue. Consequently, any observed cueing effect from the task type cue, whether on practiced or novel tasks, could be attributed only to the adjustment of working memory updating functions that were independent of task identity. However, our findings showed no significant effect of pre-cues, thereby providing no evidence for the idea that a working memory gating function can be adjusted by instruction in a fast-paced manner.

More broadly, our findings show some analogues to previous studies that highlighted the challenge of preparing for different congruency or task switching conditions based on preparatory cues (e.g. Bugg & Smallwood, 2016; Jimenez et al., 2021; Dreisbach, Haider, and Kluwe, 2002; for a review, see Braem et al., 2024). Namely, these findings similarly emphasize that adjusting task processing by only cueing stimulus- or task-agnostic control demands cannot be easily achieved. Please note that these studies employed different paradigms than ours, thus our findings regarding the working memory gating control might not be totally comparable to theirs. Compared to previous studies which typically only recruited twenty to thirty participants per experiment, we recruited more than fifty participants per experiment in two different cohorts from either Dutch- or English-speaking population. An additional meta-analysis was also conducted to further integrate our findings over these four experiments (data from 222 participants in total), and the result of both RT and accuracy demonstrated clear null effects of task type cue, clearly indicating no behavioral benefit afforded by our task type cues.

One of the potential critiques on our null findings could be that there was not enough training on the practiced tasks for a differential task configuration process to be adopted. However, a recent study (Mill & Cole, 2023) included a comparable level of training on the PRO tasks observed a clear post-training shift in neural representational dynamics during task implementation. Also in our study, we observed robust novelty costs (or practice benefits) in behavior across all four experiments, suggesting task training did work. Together, it seems unlikely that the practiced tasks were not trained enough to impose a different configuration process compared to novel tasks. However, future study could still try to include more training before testing to ensure that the task was experienced as less challenging. Potentially, the difficult nature of the PRO task may have discouraged participants to prepare in advance due to the high cognitive load. Therefore, one potential solution would be to increase the amount of training to encourage people to prepare more.

An alternative explanation may be that people can use these cues, but were simply not sufficiently motivated to do so. Along those lines, it has been found that offering extra incentives or demanding fast responses can boost the cueing effect of congruency pre-cues (Bugg et al., 2015), suggesting that pre-cueing can be effective when people were adequately motivated (see also, Kukkonen et al., 2025). This is also in line with the theory of expected value of control (Shenhav, Botvinick, & Cohen, 2013), which proposed that exerting control or not, as well as how much control should be allocated, are determined by expected payoffs of applying control, which takes into account both the cost of cognitive effort, and the expected rewards. Thus, offering rewards could be a way to encourage adjusting cognitive control in advance. Future studies could examine whether providing extra incentives can enhance the utility of pre-cues in a multi-task environment.

Apart from the cueing effect on the incoming task, we also explored the potential benefit of task type cues in switching between practiced and novel tasks. In Experiment 3, we found the participants performed more accurately when switching from practiced to novel tasks in the informative condition compared to uninformative condition, suggesting a potential benefit of task type cue in preparing to switch to novel tasks. However, this effect was absent in Experiment 4, in which we adopted exactly the same design as Experiment 3. Combining data from these two experiments again yielded a null finding. Thus, failing to replicate the cueing effect suggested the effect we observed in Experiment 3 is probably unreliable. Another reason for not putting emphasis on this finding in Experiment 3 lies in the lack of a general switch cost between practiced and novel tasks, which implies that participants did not represent the tasks in a hierarchical structure where tasks would be grouped into task types (either practiced or novel). This is in line with previous studies that only found additional switch costs in the higher order, when tasks were actually hierarchically represented (e.g. Kleinsorge & Heuer, 1999). In our study, it suggested that, even if there was a difference in task configuration processes between practiced and novel tasks, the lack of hierarchical representation based on task types (i.e. practiced versus novel) would render the task type cue being unhelpful in terms of switching since, without the hierarchical representation, switching between two practiced tasks is not so different from switching between a practiced and a novel task. Thus, one future direction could be to further explore under which conditions people would organize the tasks in a hierarchical structure based on task novelty.

In conclusion, the current study demonstrated, across four experiments, that providing pre-cues signalling whether an incoming task will be novel or practiced, without specifying task identity, did not help participants perform. This suggests that people may not be able to exploit the pre-cue by pre-adjusting the different task configuration processes between novel and practiced tasks that previous studies have proposed (e.g., Cole et al., 2010; Ruge & Wolfensteller, 2010). Notably, our findings are concordant with prior research that similarly underscored the limitations associated with instruction-based control adjustments (e.g. Jimenez et al., 2021; Abrahamse et al., 2022), highlighting important constraints of humans’ cognitive flexibility.

## Data Accessibility Statement

The word stimuli, experimental scripts, and the raw data of current study can be found on OSF (https://osf.io/fstn6).

## Footnotes

Given that there were two word stimuli in each trial, the logical rule of BOTH requires both stimuli are “True” based on the semantic rule, AT LEAST ONE requires one or two stimuli are “True”, NEITHER calls for “False” for both stimuli, FIRST refers to the upper word, and SECOND refers to the lower word, while DIFFERENT requires one “True” and the other one being “False”.

80% was used as the criterion in the preregistration. However, after data collection, it came to our attention that 80% was too stringent. If we used 80% as the criterion, then 23.33% participants had to be excluded. Thus, we lowered the threshold to 70%, which was consistently applied for all subsequent experiments. Additional analysis confirmed that the statistical conclusions still held after applying the pre-registered exclusion criterion.
